# Cognitive Testing and Piloting of the Bangla Version of the Washington Group Short Set Questionnaire on Functioning Among Adolescent Girls and Women With Disabilities in Selected Sub-districts of Bangladesh

**DOI:** 10.7759/cureus.54953

**Published:** 2024-02-26

**Authors:** Munzur E Murshid, Sanmei Chen, Namira Rahman Era, Yoko Shimpuku, Md Moshiur Rahman, Md Ziaul Islam

**Affiliations:** 1 Graduate School of Biomedical and Health Sciences, Hiroshima University, Hiroshima, JPN; 2 Health Sciences, Graduate School of Biomedical and Health Sciences, Hiroshima University, Hiroshima, JPN; 3 Maternal and Child Health, Independent General Practice, Dhaka, BGD; 4 Community Medicine, National Institute of Preventive and Social Medicine, Dhaka, BGD

**Keywords:** pilot test, cognitive test, wg-ss, bangladesh, disability

## Abstract

Introduction

This study focuses on the cognitive testing and piloting of the Bangla version of the Washington Group Short Set Questionnaire on Functioning among adolescent girls and women with disabilities in selected sub-districts of Bangladesh. The Washington Group on Disability Statistics developed the questionnaire as a tool to assess the functioning and disability status of individuals. The adaptation of this questionnaire to Bangla is crucial for capturing accurate data on the experiences of adolescent girls and women with disabilities in Bangladesh.

Materials and methods

The research employs a two-phase approach, starting with cognitive testing to ensure the linguistic and cultural relevance of the translated questionnaire. This phase involves engaging with a sample of the target population to assess the comprehension, clarity, and appropriateness of the questions. Subsequently, a pilot study was conducted in selected sub-districts to evaluate the feasibility and validity of the Bangla version of the Washington Group Short Set Questionnaire on Functioning in real-world settings. Both of the tests were conducted in March 2023.

Results

There were different types of participants with different types of disabilities. Information processing, meaning, understanding the questions, thinking, and answering speed or time were different between groups, even though they were different from person to person. The initial assessments indicate strong consistency in responses. Participants demonstrated a favorable response rate, indicating potential effectiveness for broader implementation.

Conclusion

The current study aims to contribute to disability data collection methodologies, particularly in the context of adolescent girls and women in Bangladesh. The research seeks to empower policymakers, researchers, and advocacy groups with a robust instrument of disability screening. Researchers and clinicians may rely on our accurate and validated Washington Group Short Set Questionnaire on Functioning translation into Bangla when working with adolescent girls and women with disabilities.

## Introduction

Approximately 165 million people live in Bangladesh, where 7.9% of those over the age of 15 experience functional challenges [1]. The Bangla version of the Washington Group Short Set Questionnaire on Functioning is crucial for ensuring inclusivity and accurate data collection in Bangladesh (Appendices). Localizing the questionnaire in Bangla facilitates comprehension among Bengali-speaking individuals, enabling them to express their functioning and disability status accurately. This linguistic adaptation is essential for promoting equitable healthcare, policy formulation, and resource allocation tailored to the specific needs of the persons with disabilities in Bangladesh.

The current research endeavors to bridge a critical gap in disability data collection by undertaking the cognitive testing and piloting of the Bangla version of the Washington Group Short Set Questionnaire on Functioning specifically tailored for adolescent girls and women with disabilities in selected sub-districts of Bangladesh. The Washington Group's internationally recognized tool provides a comprehensive framework for assessing functioning and disability. By delving into the cognitive testing phase, we aim to ensure the linguistic clarity and cultural appropriateness of the translated instrument. Subsequently, the piloting phase will take place in real-world settings, enabling us to evaluate the feasibility and validity of the Bangla-adapted Washington Group Short Set Questionnaire on Functioning. This research is not only an academic exercise but a crucial step towards empowering policymakers, researchers, and advocacy groups with an effective tool to understand and address the unique challenges faced by adolescent girls and women with disabilities in Bangladesh.

Objectives of the study

To assess the questionnaire's comprehensibility, relevance, and appropriateness for collecting data on disabilities among women and adolescent girls in specific sub-districts of Bangladesh.

What is a cognitive test of a questionnaire?

A cognitive test of a questionnaire assesses respondents' understanding, interpretation, and clarity of the survey's questions. This pre-testing phase aims to identify potential issues with language, cultural relevance, or comprehension, ensuring the questionnaire is clear and effective before wider implementation [2].

What is pilot testing of a questionnaire?

Pilot testing of a questionnaire involves administering the survey to a small, representative group before full-scale implementation. This trial run helps identify potential issues with the questionnaire's clarity, relevance, or format, allowing researchers to make necessary adjustments and improve the overall quality of the survey instrument [3].

## Materials and methods

Washington Group Short Set Questionnaire on Functioning

The Washington Group Short Set Questionnaire on Functioning is a widely used tool for assessing disability and functioning in population-based surveys. Comprising six core questions, it is designed to provide a simple and standardized way to collect data on disability, making it easier to compare results across different regions and time periods. The questions cover areas such as vision, hearing, mobility, cognition, self-care, and communication, helping to identify individuals who may face difficulties in various domains of life. This questionnaire is valuable for policymakers, researchers, and organizations working to understand and address disability-related issues, promoting inclusion and tailored support for individuals with disabilities [4].

The questions are [4]: 1) Do you have difficulty seeing, even if wearing glasses?; 2) Do you have difficulty hearing, even if using a hearing aid?; 3) Do you have difficulty walking or climbing steps?; 4) Do you have difficulty remembering or concentrating?; 5) Do you have difficulty (with self-care such as) washing all over or dressing?; 6) Using your usual language, do you have difficulty communicating (for example understanding or being understood by others?

Difficulty in different domains is measured using four graded response categories: cannot do at all, some difficulty, a lot of difficulty, and no difficulty. Disability was defined by the Washington Group as the inability to perform any one domain at all or with a lot of difficulty [1].

Language validity

Following both forward and backward translation, a group of academicians were given the scale translated into Bangla to evaluate each item's level of comprehension and its cultural appropriateness in Bangla. The literature mentioned the content validity criteria as 0.62 [5]. Based on these academic experts' opinions and the Lawshe approach [6], it was concluded that all the scale's items were 0.99. No item was removed.

Cognitive test of the Bangla version of the Washington Group Short Set Questionnaire on Functioning

A cognitive test of the Bangla version was conducted among 32 adolescent girls and women with disabilities. Participants were the residents of the Kurigram Sadar sub-district of Bangladesh. The test area and participants were selected purposefully based on the socio-cultural and economic character of the area. The cognitive test was conducted in the first half of March 2023.

Training of interviewers

The training of interviewers is a critical component of ensuring the consistency of data collection. The principal investigator provided training to the interviewers. In the training, they received instructions on the questionnaire's administration and were equipped with the skills to communicate effectively with women and adolescent girls with disabilities. Through the training, interviewers will be well-versed in the questionnaire's content and purpose, as well as their cultural sensitivity to the context in which they will be working. They received training on sensitivity to ensure that interviewers approach their work with empathy and respect for the participants. Furthermore, the training included practice sessions and mock interviews to familiarize interviewers with the potential challenges they might encounter in the field. This rigorous preparation helped them contribute to the success of the study, yielding accurate and meaningful data on disability among this specific demographic in Bangladesh.

Pilot testing of the Bangla version of the Washington Group Short Set Questionnaire on Functioning

A pilot test of the Bangla version was conducted among 70 adolescent girls and women with disabilities. Participants were the residents of Khulna Sadar sub-district of Bangladesh. The test area and participants were selected purposefully based on the socio-cultural and economic character of the area. The pilot test was conducted in the second half of March 2023.

Ethical considerations

The Institutional Review Board of the National Institute of Preventive and Social Medicine (NIPSOM), Dhaka, Bangladesh, has provided IRB approval reference No. NIPSOM/IRB/2023/07, dated February 9, 2023. During recruitment, participants provided their informed written consent. Before receiving informed written permission, participants were briefed about the study objectives, procedures, and measures to ensure privacy and anonymity. They are also well-versed in the fact that they could revoke this research regardless of the survey period. The legal guardian provided informed consent on the participant's behalf when she was under 18.

## Results

Cognitive test outcomes

Summarizing Notes

Once completed, the interview was reviewed as soon as possible to ensure a good memory of what was said. Additional notes and summary points were made on the notepads of the interviewers.

Summary Comments on Interview Context

Example 1: The respondent was a homemaker. Her age was 35. The woman was physically impaired. She was quite eager to take part in the research. She was having trouble understanding the difficulties she encountered regularly because of her physical disability though. After listening to the interviewer's examples, she finally answered the questions accurately.

Example 2: The 30-year-old interviewee was a housewife. She suffers from a partial hearing impairment. Her interview took 36 minutes to finish. The interviewer made an effort to talk more loudly and slowly. He also makes sure the space is properly lit. People with disabilities can occasionally lip-read to understand what others are saying. The interviewee requested to hear every question many times, and she took her time responding to each one. But she didn't seem tense at all. Her mother-in-law and husband were present when she was interviewed at her house. The interviewer used some probing questions to open up her thought process. For example, the interviewer asked her, ‘If two people talk with each other in their usual voices in a room, do you understand their conversation’. She said, ‘I have a lot of trouble understanding conversations between two people, even in a quiet room.' 

Example 3: Another respondent consented to take part in the interview. But she struggled to speak, so she wasn't very good at responding to most questions. Her age was 33. The lady struggles to carry on long discussions, has problems speaking in public, and has a partial speech disability. She stated that she has had communication difficulties since her childhood. She said she found it difficult to socialize. Her speech impediment makes her uncomfortable in social situations. Her tongue was twisting a lot while she spoke, which made it hard for her to pronounce words clearly.

Example 4: A 32-year-old respondent with partial visual impairment replied well. Her reply was really intelligent and well-spoken. The interviewee provided answers on the spot. She did a wonderful job responding to the queries.

Participants' Understanding of Survey Questions

Ensuring the validity of research findings significantly relies on the respondents' understanding of the survey questions. In order to make sure that the survey questions are understandable, culturally appropriate, and sensitive to the distinct experiences of women and adolescent girls with disabilities in the selected sub-districts of Bangladesh, researchers attempted to understand how respondents interpret the questions in this study. By applying cognitive testing researchers try to find potential challenges in comprehension, language difficulties, or cultural nuances that could affect respondents' capacity to give correct and insightful answers.

In our testing, a group of participants had visual and hearing difficulties, but they did not use any visual or hearing aids. In that case, the interviewer used some probing or exemplary sentences along with the set questions.

Set questions: 1. Do you have difficulty seeing, even if wearing glasses?; 2. Do you have difficulty hearing, even if using a hearing aid(s)?; 3. Probing or exemplary questions or sentences for a person with a hearing disability and not using a hearing aid such as "Are you able to understand what the two persons in your room are saying?"; 4. Probing or exemplary questions or sentences for a person with a visual disability and not using eyeglasses: a. "Can you read the Quran with bare eyes?", b. "Can you thread a needle with your bare eyes?"

The researchers did not exclude ‘even if wearing glasses’ or ‘even if using a hearing aid(s)’ phrases because the ‘Washington Group Short Set Questionnaire on Functioning’ is an established questionnaire. Researchers might overlook individuals who are susceptible to hearing or vision impairments if these phrases are removed. Only the seeing and hearing domains were given consideration for the inclusion of assistive devices, as the usage of glasses or hearing aids can frequently readily overcome impairments in these domains [7]. Some study participants were using visual or hearing aids. Among the 30 participants in the cognitive test, 10 were using hearing aids, 7 were using walking aids, and 3 were eyeglass users.

Information Processing by Respondents

There were different types of participants with different types of disabilities. Information processing, meaning, understanding the questions, thinking, and answering speed or time were different between groups, even though they were different from person to person. Persons with partial hearing disabilities needed more repetition of questions, and they needed to hear the questions loudly. Persons with partial visual disabilities needed an interview place with good lighting so that they could do a lip reading of the interviewer properly. Adolescent girls with disabilities needed their parent's cooperation in understanding and responding to questions. Women with advancing age and less education or community exposure needed more probing questions or examples to answer the survey questions.

Pilot Test Outcomes

Pilot test response statistics: In case of question one 'Do you have difficulty seeing, even if wearing glasses?' Out of the 70 participants, 55 indicated they had "no visual difficulty," 3 said they had "some difficulty," and 12 said they had "a lot of difficulty" seeing, even if they wear eyeglasses (Table 1).

**Table 1:** Participants response to Washington Group Short Set Questionnaire on Functioning Participants response summary

	Vision	Hearing	Mobility	Cognition	Self-care	Communication
No difficulty	55	59	23	62	33	60
Some difficulty	3	3	4	6	34	5
A lot of difficulty	12	8	41	2	3	5
Cannot do at all	-	-	2	-	-	-
Total	70	70	70	70	70	70

For the second question, 'Do you have difficulty hearing, even if using a hearing aid?' Out of the 70 individuals who took part in the study, 59 said they had "no hearing difficulty," three said they had "some difficulty," and eight said they had ‘a lot of difficulty’ in hearing even if they use hearing aids (Table 1).

In the case of the third question 'Do you have difficulty walking or climbing steps?' Among the 70 respondents, 23 respondents replied they have ‘no walking difficulty’, four respondents replied they have 'some difficulty’, 41 respondents replied they have ‘a lot of difficulty’ in walking, and two participants stated they ‘cannot walk’ at all (Table 1).

In the case of the fourth question 'Do you have difficulty remembering or concentrating? Six respondents indicated they had "some difficulty," two respondents indicated they had "a lot of difficulty," and 62 respondents out of the 70 respondents said they had "no difficulty" remembering or concentrating (Table 1).

For the fifth question, 'Do you have difficulty with self-care, such as washing all over or dressing?', among the study participants (70 respondents), 33 respondents replied they have ‘no difficulty’ in self-care, such as washing all over or dressing; 34 respondents replied they have 'some difficulty'; and 3 respondents replied they have ‘a lot of difficulty’ in self-care (Table 1).

In the case of the sixth question 'Using your usual language, do you have difficulty communicating for example understanding or being understood?', out of the 70 respondents, 60 said they had "no difficulty" in communicating using their usual language, five said they have "some difficulty," and five more said they have "a lot of difficulty" (Table 1).

The pilot study on the ‘Washington Group Short Set Questionnaire on Functioning’ revealed promising results. Initial assessments indicate strong consistency in responses. Participants demonstrated a favorable response rate, indicating potential effectiveness for broader implementation. Nonetheless, ongoing validation efforts are imperative to fine-tune its applicability across diverse contexts and populations, ensuring its robustness and effectiveness in capturing comprehensive functional status information.

## Discussion

The cognitive testing and piloting of the Bangla version of the Washington Group Short Set Questionnaire on Functioning among Adolescent Girls and Women with Disabilities in selected sub-districts of Bangladesh yielded valuable insights into the tool's applicability and effectiveness in this context. Our findings highlight the importance of linguistic and cultural nuances.

During the cognitive testing phase, we observed that certain questions required clarification. The piloting phase allowed us to assess the feasibility of administering the questionnaire to the target population. The feedback from participants provided essential information on the comprehensibility of the questionnaire, helping to enhance its overall clarity and relevance. Furthermore, insights gained from the piloting process facilitated adjustments to the administration process, contributing to the optimization of data collection efficiency.

In our current study, a cognitive test was applied to 30 women and adolescent girls with disabilities from the Kurigram sub-district of Bangladesh. The study participants were selected purposefully, considering socio-economic conditions and study objectives. Bangladesh has 64 districts. Each district is composed of several sub-districts. Districts and sub-districts are administrative areas based on their geographic and administrative boundaries [8]. A study conducted by Miller et al. (2011) conducted cognitive tests among 1,290 respondents. Respondents were selected by convenience sampling techniques. Participants were selected from 15 countries in Central and South America, Asia, and Africa [9].

According to Miller et al. (2011), different study locations' linguistic differences created some difficulties. Some nations had little to no prior experience carrying out this kind of assessment. They were lacking qualified cognitive interviewers (qualitative interviewers). Managing all of the fieldwork by a single research team was difficult [9].

In the current study, all respondents were native Bangla speakers. Participants' socio-demographics were similar. In the study team, we have a qualitative study expert and researcher with remarkable disability field exposure with international organizations. Three of the researchers were from Bangladesh. In our study, one team was able to oversee all of the research activities.

Hancock et al. (2023) stated there are several cut-off categories to decide disability status. These are: a) If anyone stated he/she has 'some difficulty’ at any domain (vision, hearing, mobility, cognition, self-care, communication); (b) a person with 'some difficulty’ in two or more domains, a person with 'a lot of difficulty,' or a person who stated 'cannot do at all' at any domain; (c) person with ‘a lot of difficulty’ in one or more domains, or he or she stated ‘cannot do at all’ in one or more domains; (d) if any person stated ‘cannot do at all’ in one or more domains [10]. In the current study, cut-off category three was followed. Along with disability screening purposes, this cut-off category can also be used to identify long-term needs and income support needs [10].

UN-ESCAP (United Nations Economic and Social Commission for Asia and the Pacific) researchers applied different sampling techniques in their study to select households to survey. They applied systematic sampling for Cambodia, the census area principle was applied for Kazakhstan, purposive sampling for the Maldives, a two-stage sampling method for Mongolia, criteria-based purposive sampling for the Philippines, and cluster sampling for Sri Lanka [11]. In the current study, the researcher selected study areas considering the socio-economic condition of the area and available resources (funding, manpower, etc.) for the study.

Strength of the study

This is the first-reported cognitive test and pilot test of the Bangla version of the Washington Group Short Set Questionnaire on Functioning. The research team has in-depth knowledge and experience on respective issues (disability, research methodology, Bangladesh contexts).

Limitations of the study

The study focuses only on women and adolescent girls with disabilities in selected sub-districts of Bangladesh, limiting the ability to generalize the findings to the wider population of women and adolescent girls with disabilities. To reduce the limitations in the future, based on available funding, more study sites will be recruited with larger sample sizes, and the questionnaire can be tested among different groups of participants (male, female, adolescent, elderly, young, etc.).

## Conclusions

In Bangladesh, different bodies (the government and development partners) use different tools to measure disability. The country needs a specific tool to screen for disability. This is the first published native version of the tool. The cognitive testing and piloting of the Bangla version of the ‘Washington Group Short Set Questionnaire on Functioning’ among adolescent girls and women with disabilities in selected sub-districts of Bangladesh yielded significant insights. The study demonstrated the questionnaire's relevance and effectiveness in capturing the unique experiences and challenges faced by this demographic. The findings underscore the importance of utilizing culturally adapted tools to accurately assess the functioning of individuals with disabilities, thereby providing a foundation for targeted interventions and policy formulation to address the specific needs of adolescent girls and women with disabilities in Bangladesh. Researchers, clinicians, and other healthcare professionals can use this Bangla version for disability screening purposes.

## Figures and Tables

**Figure 1:**
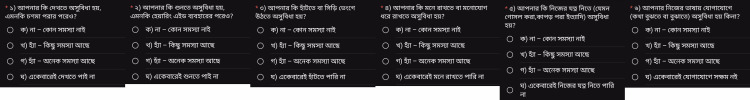
Bangla translation of the Washinton Group Short Set Questionnaire on Functioning Washington Group Short Set - Bangla
